# Extracellular Proteolysis of Apolipoprotein E (apoE) by Secreted Serine Neuronal Protease

**DOI:** 10.1371/journal.pone.0093120

**Published:** 2014-03-27

**Authors:** Irfan Y. Tamboli, Dongeun Heo, G. William Rebeck

**Affiliations:** Department of Neuroscience, Georgetown University, Washington DC., United States of America; University of S. Florida College of Medicine, United States of America

## Abstract

Under normal conditions, brain apolipoprotein E (apoE) is secreted and lipidated by astrocytes, then taken up by neurons via receptor mediated endocytosis. Free apoE is either degraded in intraneuronal lysosomal compartments or released. Here we identified a novel way by which apoE undergoes proteolysis in the extracellular space via a secreted neuronal protease. We show that apoE is cleaved in neuronal conditioned media by a secreted serine protease. This apoE cleavage was inhibited by PMSF and α1-antichymotrypsin, but not neuroserpin-1 or inhibitors of thrombin and cathepsin G, supporting its identity as a chymotrypsin like protease. In addition, apoE incubation with purified chymotrypsin produced a similar pattern of apoE fragments. Analysis of apoE fragments by mass spectrometry showed cleavages occuring at the C-terminal side of apoE tryptophan residues, further supporting our identification of cleavage by chymotrypsin like protease. Hippocampal neurons were more efficient in mediating this apoE cleavage than cortical neurons. Proteolysis of apoE4 generated higher levels of low molecular weight fragments compared to apoE3. Primary glial cultures released an inhibitor of this proteolytic activity. Together, these studies reveal novel mechanism by which apoE can be regulated and therefore could be useful in designing apoE directed AD therapeutic approaches.

## Introduction

Alzheimer's Disease (AD) is the most common cause of elderly dementia and is characterized by extracellular deposition of amyloid β-peptide (Aβ) plaques in brain [1], [2]. Sequential cleavage of amyloid precursor protein (APP) by β- and γ-secretase to generate Aβ is believed to initiate a pathogenic cascade that leads to AD [3], [4]. The strongest genetic risk factor for late onset AD is APOE [5], [6]. Being heterozygous or homozygous for the APOE-ε4 allele confers a 3 or 10 fold increase in AD risk, respectively. Although exact numbers vary, approximately 50% of all AD patients have at least one APOE ε4 allele and inheritance of the APOE ε4 allele significantly lowers age of onset [7]–[9].

The apolipoprotein E (apoE) protein is a 34-kDa glycoprotein associated with lipoproteins in periphery and brain. ApoE exists in humans as three major isoforms (apoE2, apoE3, apoE4) that differ from each other at amino acid positions 112 and 158 [10], [11]. ApoE4 is associated with higher plaque load in human AD patients and AD mouse models [12]–[14]. ApoE plays a potential role in Aβ aggregation and apoE4 has been suggested to be responsible for increased levels of neurotoxic Aβ oligomers. ApoE modulates clearance of Aβ across blood-brain barrier as well as its degradation in lysosomal compartments by aiding its endocytosis; apoE4 is believed to be less efficient in these functions [15]–[18]. Perivascular drainage of apoE also influences clerance of Aβ and thereby impacts the development of cerebral amyloid angiopathy [19], [20].

The level of APOE mRNA in brain is second only to liver. Astrocytes are the primary source of apoE in brain, although microglia and neurons have been shown to synthesize apoE under certain circumstances [21]. ApoE is secreted by astrocytes into the extracellular space and is detected in cerebrospinal fluid (CSF) as a component of high density lipoproteins. Binding of apoE to members of the low density lipoprotein receptor (LDLR) family and its subsequent endocytosis are necessary for efficient uptake of lipoproteins by neurons, which supports neuronal maintenance, growth, and repair [18], [22], [23].

APOE genotype influences apoE protein levels in brain, with apoE4 levels lower than those of apoE3 [23]–[27]. This has been attributed to lower stability and increased turnover of apoE4, although there are no differences in fractional turnover rates of apoE3 and apoE4 in human CSF [28]. Multiple proteolytic enzymes, such as cathepsins, calpains and chymotrypsin-like protease have been suggested to mediate apoE proteolysis to generate neurotoxic fragments [29]–[32]. ApoE fragments are found in AD brains associated with amyloid plaques and neurofibrillary tangles (NFTs) [33]–[35]. Differential proteolysis of apoE4 and toxicity exerted by its cleavage products may play an important role in AD pathology. However, subcellular location of apoE cleavage is not well established, with some support for intraneuronal proteolysis [32]. In this study we tested for the presence of secreted apoE proteolytic activities.

## Materials and Methods

### Materials

Human recombinant (rec) neuroserpin, purified human plasma α1-ACT, bovine pancreatic α-chymotrypsin, rec apoE3 and apoE4, purified cathepsin G from human leukocytes, purified thrombin from human plasma, and argotroban, were all obtained from Sigma. VLDL-apoE purified from human plasma was from rpeptide. Cathepsin G inhibitor was obtained from Calbiochem. PMSF and protease inhibitor mix were from ROCHE. D6E10 anti apoE antibody raised against apoE amino acids 141–160 was from Cell Signaling.

### Preparation of rec apoE3 and apoE4

Rec human apoE3 and apoE4 were obtained by overexpression in Escherichia coli strain BL21-CodonPlus using pET32a vectors with thioredoxin as the fusion partner (Novagen). Vectors containing human cDNA for either apoE3 or apoE4 were kindly provided by Karl Weisgraber. The apoE-thioredoxin complex was obtained by sonicating the cells and removing debris by centrifugation at 32 000 rpm for 20 min using a TLA-100.4 rotor (Beckman). Thioredoxin was cleaved from the apoE with thrombin, and apoE was isolated by gel permeation chromatography on a column of Sephacryl S-300 (Amersham).

### Primary neuronal cultures and glia cultures

Primary hippocampal and cortical neuronal cultures were prepared from Sprauge Dawley rats at embryonic day 18–19 embryos plated density (∼750 cells mm^−2^). Cultures were grown in Neurobasal medium supplemented with B27 (Gibco). Primary glia were prepared from Sprauge Dawley rats at embryonic day 18–19 embryos. Briefly, brain tissue was homogenized sequentially through 19G, 21G and 23G needles in HBSS and cells were plated in DMEM containing 10% FBS. Medium was changed every second day. Animal handling and primary rat neuron and glia culture preparation were carried out in accordance with NIH guidelines for the care and use of laboratory animals, and were reviewed and approved by the Georgetown University Animal Care and Use Committee (protocol# 13-018-100076). Rats were euthanized humanely using CO2 chamber.

### Immunoblotting

15% SDS-PAGE or NuPAGE (Invitrogen) gels followed by western immunoblotting were used for apoE proteolysis analysis.

### LC-MS analysis

Excised pieces of silver stained gels were destained, dried, alkylated using 55 mM iodoacetamide and later rehydrated, followed by In-Gel digestion using 250 ng mass-spectrometry grade trypsin (Promega). Following digestion the reaction mixture was acidified to 1% tri-flouroacetic acid and dried to 5 ul volume which was loaded to a 2 cm×75 um I.D. trap column of Easy nanoLC II HPLC (Thermo) for LC-MS/MS analysis. The LC was interfaced to a dual pressure linear ion trap mass spectrometer (LTQ Velos, Thermo Fisher) via nano-electrospray ionization. An electrospray voltage of 2.0 kV was applied to a pre-column tee. The mass spectrometer was programmed to acquire, by data-dependent acquisition, tandem mass spectra from the top 15 ions in the full scan from 400–1400 m/z. Dynamic exclusion was set to 30 s. Data processing and library searching was performed on Amazon Web Services-based cluster compute instances using the Proteome Cluster interface. All searches required semi-style tryptic cleavage, up to 1 missed cleavage, fixed modification of cysteine alkylation and variable modifications of methionine oxidation. XML output files were parsed and non-redundant protein sets determined using in-house scripts.

### Zymography

Zymogram gels were obtained from Invitrogen and used according to manufacturer's instructions. Briefly, gels were run under non-reducing conditions and incubated in renaturing buffer to allow reactivation of enzymatic activity, followed by incubation in developing buffer for 24 h.

### Extracellular apoE proteolysis assay

Cell free conditioned medium was collected from DIV18-DIV22 primary rat hippocampal (HPC) or cortical (CTX) neurons. Glial serum-free conditioned medium was collected at DIV 14 when primary glial cells were washed twice with PBS, incubated with serum free DMEM for 4 h and then incubated with fresh DMEM for 24 h. 2 ug rec apoE was incubated with 100 ul final volume of either individual or mixed conditioned medium for the indicated time periods. All inhibitors used were diluted to 1× concentrations from 100× stocks.

### Human brain tissue

Human brain frontal cortex tissue was obtained from the Department of Pathology, Johns Hopkins University, Baltimore, MD (Table S1). Tissue was homogenized in RIPA buffer (1% Igepal, 0.5% Na-deoxycholate, 0.1% SDS, 50 mM Tris-HCl, pH 8). Pellets obtained were further sonicated in RIPA buffer and resuspended in reduced 4× NuPAGE LDS sample buffer (invitrogen). Samples obtained were used for detection of insoluble apoE. Prior Institutional Review Board consent was obtained from Georgetown Universities IRB committee (IRB # 03-148).

### Statistics

Immunoblot X-ray films were scanned and images were analyzed using ImageJ software. Statistical analysis of three independent experiments (n = 3) was carried out using Student's t-tests. Asterisk (*) indicates significant p value in the range of 0.001 to 0.05. Error bars represent standard deviations.

## Results

### An extracellular protease secreted by hippocampal neurons cleaves apoE4 more than apoE3

Previous studies suggested a critical role of a neuron specific protease in apoE cleavage [21]. To study the contribution of neurons in apoE proteolysis, we incubated recombinant (rec) apoE4 for various time periods with primary rat hippocampal neurons. The culture medium and cell lysates were analyzed at different time points for the presence of apoE and any lower molecular weight apoE fragments. Over time, we observed a steady decrease in levels of total apoE, together with a corresponding appearance of lower molecular weight fragments in conditioned medium (Figure S1). Levels of apoE and apoE fragments in cell lysates were relatively low (Figure S1). Coomassie stained gel of both purified rec apoE3 and apoE4 showed a single band at 34 kDa (Figure S2).

**Figure 1:**
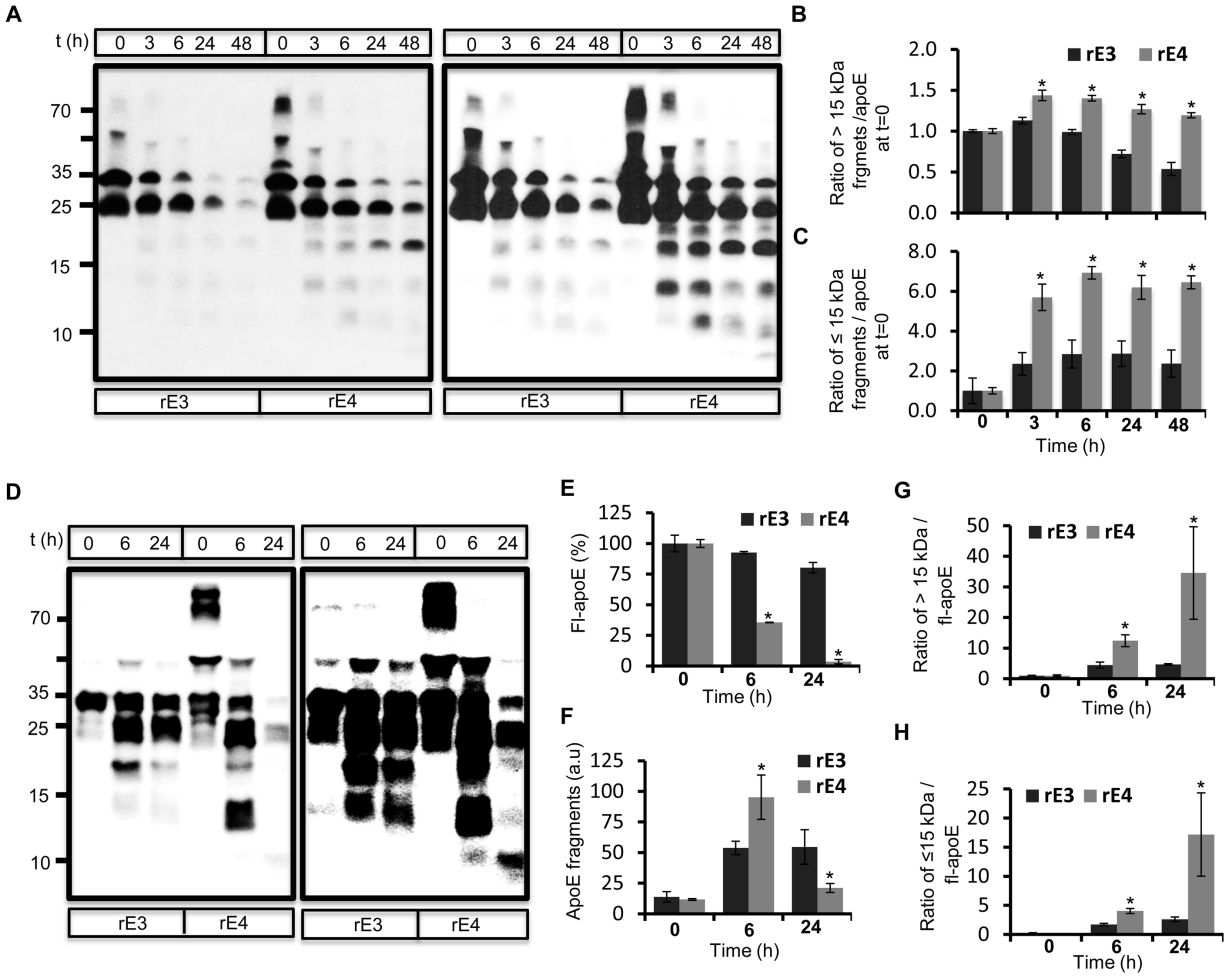
*Ex-vivo* degradation of rec apoE in conditioned medium obtained from rat primary hippocampal neuron cultures. A–C) Conditioned medium was obtained from DIV 22 rat primary hippocampal neurons, and rec apoE3 (rE3) and apoE4 (rE4) (2 ug) were incubated with conditioned medium for indicated times at 37°C. Degradation of apoE at each time point was analyzed by western immunoblotting (A). Longer exposure of same blot is shown on the right panel. The levels of apoE fragments greater than 15 kDa (B) and lesser than 15 kDa (C) compared to apoE at t = 0 were determined using ImageJ. D–H) Commercially obtained rE3 and rE4 was incubated with hippocampal conditioned medium for indicated time periods followed by analysis using western immunoblotting (D). Longer exposure of same blot is shown on the right panel. Levels of full length apoE (E), total apoE fragments (F) and ratio of apoE fragments greater than 15 kDa (G) and lesser than 15 kDa (H) to full length apoE levels were quantified using ImageJ.

The gradual shift towards lower molecular weight fragments as well their subsequent turnover with longer incubation periods in conditioned medium indicated stepwise proteolysis of apoE by hippocampal neurons. In order to test if a secreted enzyme activity was responsible for apoE proteolysis, we analyzed the metabolism of rec apoE in cell free conditioned medium collected from hippocampal neuronal cultures. As in the cellular assays, both apoE3 and apoE4 underwent stepwise proteolysis to generate similar fragments (Figure 1*A*). We calculated the rate of proteolysis by measuring the ratio of apoE fragments greater than 15 kDa to initial apoE levels (Figure 1*B*), as well as of fragments 15 kDa and lower to initial apoE levels (Figure 1*C*). More fragments were generated from apoE4 at all time points compared to apoE3. The decrease in levels of full length apoE was similar for both apoE3 and apoE4, although there was also a reduction of full length apoE3 in control non-conditioned medium (not shown). Therefore, we also analyzed proteolysis of rec apoE obtained from a commercial source (Figure 1*D*). We confirmed that both apoE3 and apoE4 were were stable in control non-conditioned culture medium (Figure S3). ApoE4 was metabolized much more rapidly than apoE3 in neuronal hippocampal conditioned medium (Figure 1*D, 1E*). Enhanced metabolism of apoE4 was further supported by increased levels of fragments at 6 h and their efficient turnover by 24 h (Figure 1*D, 1F*). The ratio of apoE fragments to full length apoE was also higher for apoE4 than apoE3 both at 6 h and 24 h (Figure 1*G, 1H*). These data show that both apoE3 and apoE4 undergo cleavage by a secreted neuronal protease, and that apoE4 proteolysis generates higher levels of proteolytic fragments. We also confirmed cleavage of apoE associated with very low density lipoproteins (VLDL-apoE) isolated from human plasma in hippocampal conditioned medium (Figure S4).

Although very efficient apoE proteolytic activity was present in hippocampal conditioned medium, the rate of apoE proteolysis varied greatly in conditioned medium obtained from primary neuronal cultures prepared at different times. This variation contributed to slightly different patterns of apoE proteolysis with respect to time in each experiment. Experiments which involved comparisons between apoE isoforms and proteolytic activities were performed using the same conditioned medium.

### ApoE metabolism by a serine protease

**Figure 2:**
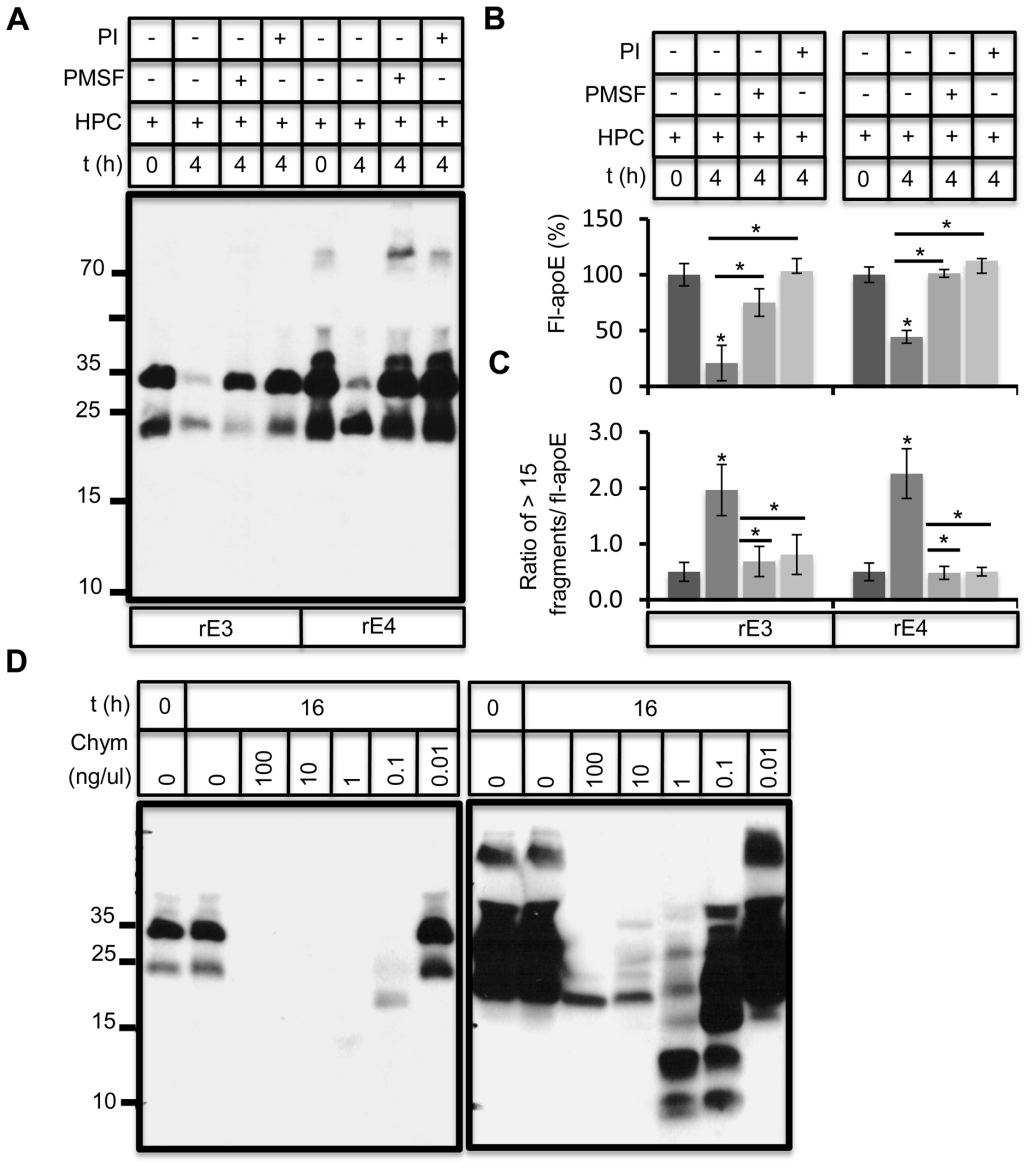
Inhibition of apoE degradation by PMSF and protease inhibitor mix. A) rE3 and rE4 proteins were incubated with DIV20 hippocampal medium for 4 hours in the absence (ctrl) or presence of 10 ug/ml PMSF or 1× complete protease inhibitor mix (PI). ApoE and its proteolytic fragments were later analyzed by immunoblotting. B, C) Full length apoE levels (B) and ratio of apoE fragments greater than 15 kDa to full length apoE (C) were measured using ImageJ. D) rE4 was incubated with indicated concentrations of α-chymotrypsin (chym) for 4 hours and generated proteolytic fragments of apoE were analyzed by western immunoblotting. A longer exposure to highlight smaller apoE fragments in shown in the right panel.

To characterize the protease responsible for apoE proteolytic activity, we performed the apoE proteolysis in the presence of different protease inhibitors. Incubation of primary hippocampal neurons with 10 uM leupeptin or conditioned medium with 10 uM EDTA did not affect apoE proteolysis (data not shown). However, apoE proteolysis was significantly impaired by a complete protease inhibitor mix (Roche) and by the serine protease inhibitor PMSF (Figure 2*A, B*), suggesting a role of a chymotrypsin like protease. The ratio of apoE fragments to full length apoE increased upon 4 hrs of proteolysis in the absence, but not in the presence of these protease inhibitors (Figure 2*C*). Incubation of rec apoE with purified bovine α-chymotrypsin at various concentrations resulted in apoE cleavage and subsequent degradation (Figure 2*D*). Importantly, the pattern of apoE fragments generated resembled the pattern of fragments generated in neuronal conditioned medium.

**Figure 3:**
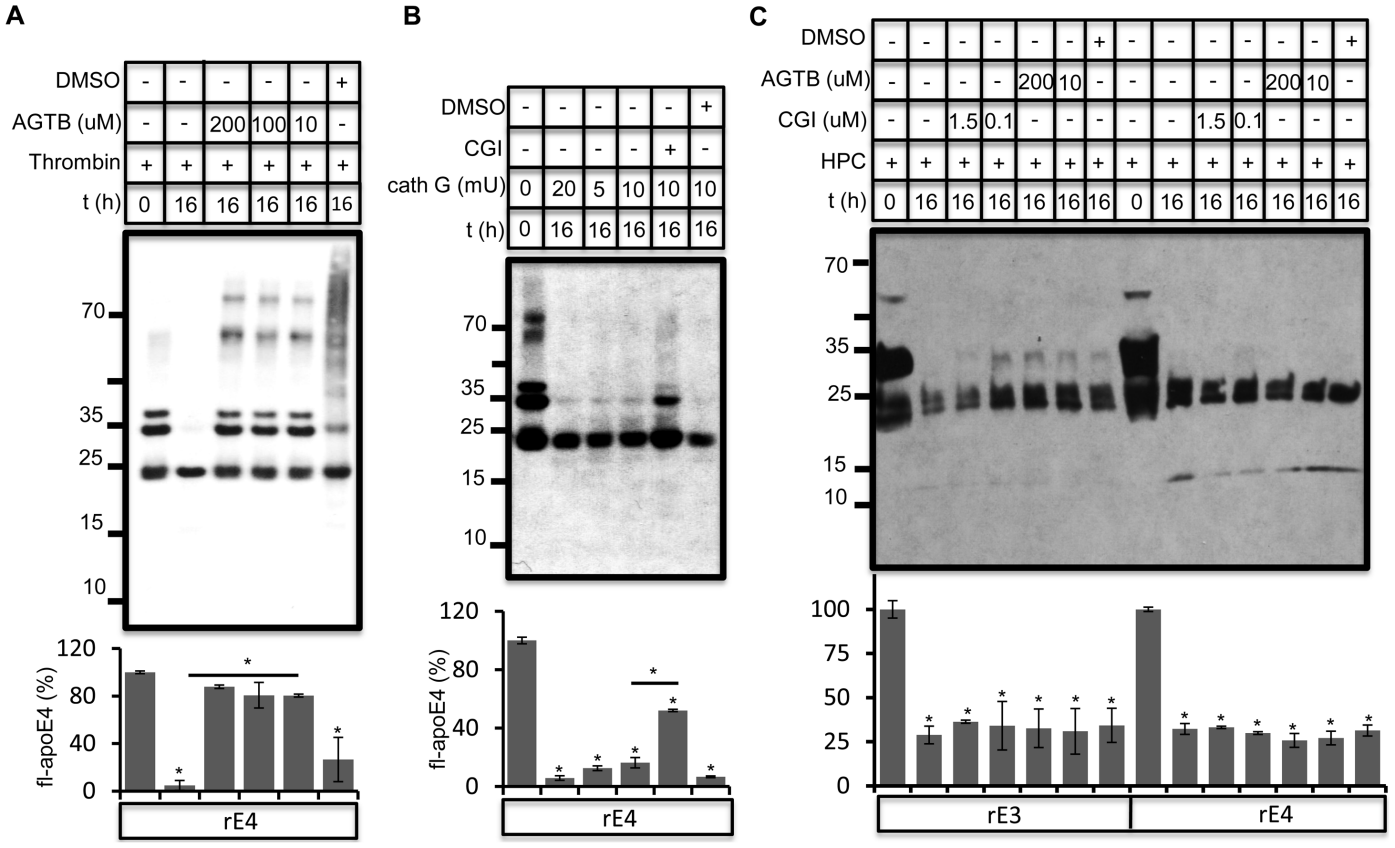
Thrombin and cathepsin G do not contribute to apoE proteolysis in conditioned neuronal medium. A) Rec apoE4 (2 ug) was incubated together with purified human thrombin (5 U) in presence and absence of direct thrombin inhibitor argotroban (AGTB) at indicated concentrations for 16 h at 37°C. Since AGTB was dissolved in DMSO, DMSO control was included. AGTB was very potent in inhibiting thrombin mediated degradation of apoE. B) Rec apoE4 (2 ug) was incubated together with purified cathepsin G (cath G) at indicated concentrations in absence and presence of 100 nM cathepsin G inhibitor (CGI) for 16 h. ApoE proteolysis was analyzed by immunoblotting. C) Rec apoE3 and apoE4 (2 ug) were incubated with conditioned hippocampal medium for 16 h at 37°C in presence and absence of thrombin inhibitor, AGTB and cathepsin G inhibitor, CGI at indicated concentrations. ApoE was analyzed by western immunoblotting. Full length apoE levels were quantified as above.

In addition to inhibiting chymotrypsin and related proteases, PMSF also inhibits thrombin and cathepsin G. Thrombin inhibitor AGTB inhibited apoE cleavage by purified thrombin efficiently (Figure 3*A*, Figure S5), while cathepsin G inhibitor CGI was partially effective in blocking cathepsin G (Figure 3*B*) [36], [37]. However, CGI and AGTB both failed to inhibit apoE proteolysis in hippocampal conditioned medium (Figure 3*C*).

**Figure 4:**
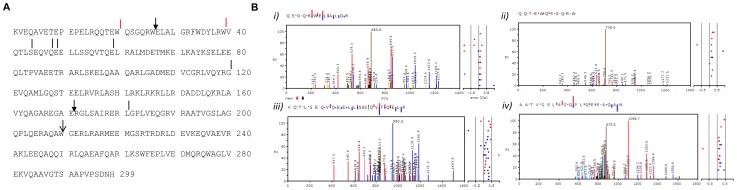
Detection of chymotrypsin cleavage sites in apoE3 using mass spectrometry. A) Representation of full length apoE3 protein sequence and observed proteolytic sites in ∼25, 20 and 17 kDa fragments generated upon incubation of rE3 in hippocampal conditioned medium. Two common cleavages sites at the carboxy side of W-20 and W-39 are indicated by red lines. Additional cleavage site in the ∼25 kDa apoE fragments at W-210 is indicated by arrow. Additional 6 cleavage sites in ∼20 kDa fragments are indicated by black lines, whereas additional two cleavage sites at W-26 and E-171 in ∼17 kDa fragments are marked by arrows with solid heads. Amino acid position is indicated by numbers. B) Annotated tandem mass spectra of identified peptides generated upon cleavage by chymotrypsin like protease at W-20 (*i*), W-26 (*ii*) W-39 (*iii*) and W-210 (*iv*).

Next, we carried out LC-MS analysis of apoE fragments. Rec apoE3 was incubated with hippocampal conditioned medium for 24 h and silver stained apoE3 fragment bands (∼25, ∼20 and ∼17 kDa) were cut from the gel, followed by trypsin digestion. Tandem MS identification of peptides confirmed the presence of apoE fragments generated by a chymotrypsin like protease (Figure 4). Control apoE3 was also analyzed by the same approach. A schematic representation of cleavage sites appearing specifically after apoE3 incubation in conditioned medium is shown in Figure 4A. Red lines represent cleavage sites common in all three molecular weight fragments, whereas arrows, black lines, and arrows with solid heads indicate cleavage sites present in ∼25, 20 and 17 kDa bands, respectively (Figure 4*A*). Cleavage at the C-terminal side of tryptophan residues (W20, W39 and W210) in the high molecular weight apoE fragments strongly suggests a a role of chymotrypsin like activity in the initial cleavage. Subsequent cleavages at other sites including the C-terminal side of L181 and W26 in the ∼20 kDa and 17 kDa apoE fragments, respectively, suggests further metabolism of apoE by a chymotrypsin like activity and other proteases (Figure 4*A*). Mass spectrometry chromatograms (Figure 4*B*) confirmed the presence of chymotrypsin cleavage sites in peptides generated after tryptic digestion.

### Inhibition of extracellular apoE proteolysis by a glial secreted factor

**Figure 5:**
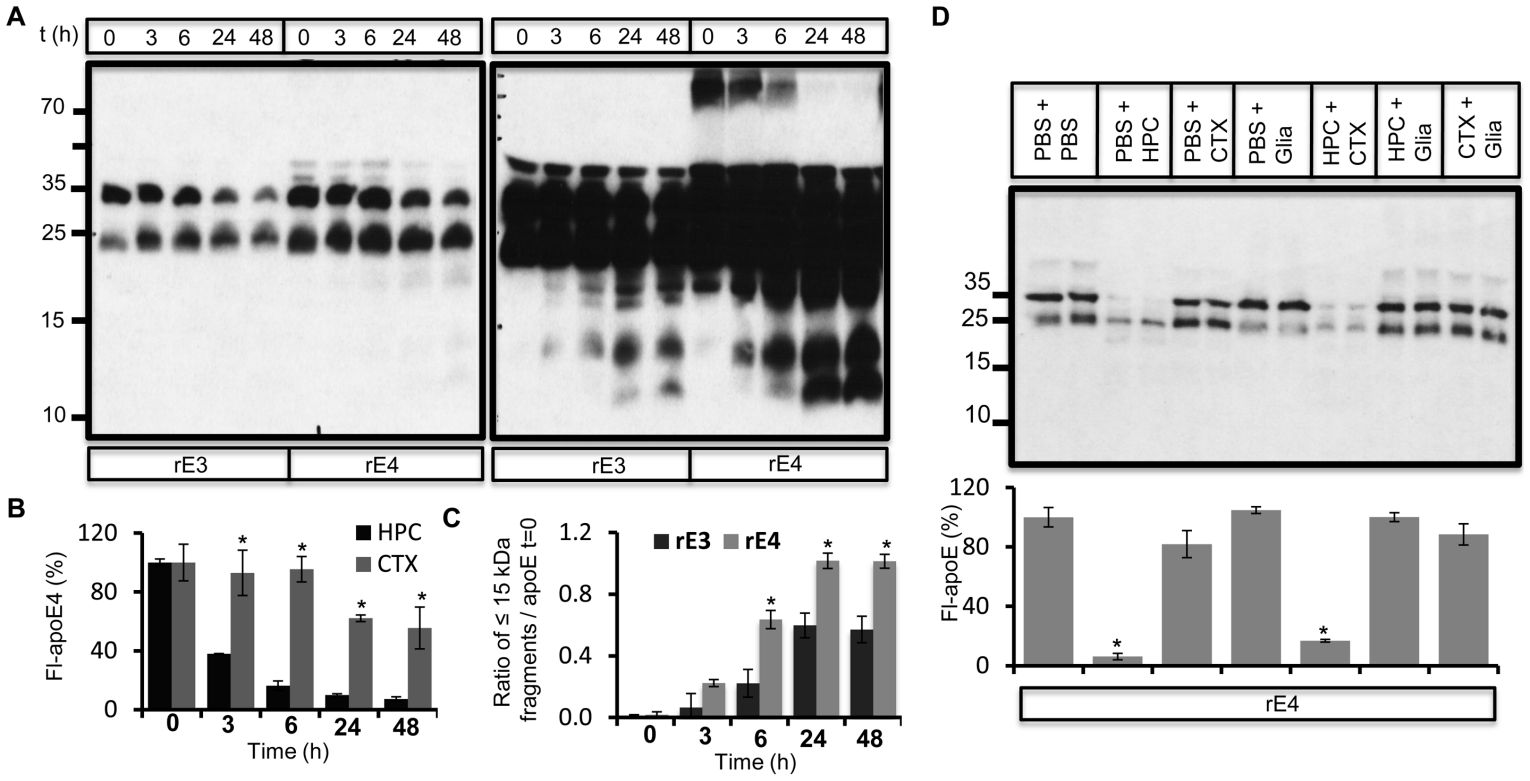
Inhibition of apoE proteolysis in presence of glia conditioned medium. A) rE3 and rE4 proteins were incubated with conditioned media obtained from primary cortical neurons (DIV22) for indicated times. Full length apoE and its proteolytic products were analyzed by western immunoblotting. Longer exposure of same blot is shown in the right panel. B) Comparison of time dependent changes in full length apoE4 levels upon incubation in cortical (CTX) and hippocampal (HPC, from fig. 1A) conditioned medium. C) Ratio of apoE fragments lesser than 15 kDa to initial apoE levels was quantified using ImageJ. D) Conditioned medium obtained from primary rat hippocampal (HPC), cortical (CTX) and glia cells were mixed with equal volumes of PBS or each other as indicated. 2 ug rE4 was incubated with these conditioned medium for 6 h at 37°C, followed by western immunoblot analysis of apoE. Full length apoE levels were quantified as indicated.

In order to asses the cellular specificity of the apoE degrading protease, we compared apoE proteolysis in conditioned medium obtained from hippocampal or cortical neurons and from mixed glia cultures. ApoE proteolysis was less efficient in cortical conditioned medium compared to hippocampal conditioned medium. Within 48 h, only about 50% of the initial apoE was metabolized by conditioned medium from cortical neurons (Figure 5*A, 5B*), whereas about 80% of the initial apoE was metabolized by medium from hippocampal neurons (Figure 1*A*, 5*B*). Similar to hippocampal cultures, media from cortical cultures produced a significantly higher ratio of apoE4 fragments to initial apoE levels (Figure 5*C*). Levels of apoE fragments increased until 48 h, and very low molecular weight fragments (lower than 10 kDa) were absent, indicating slower proteolysis. We did not observe any significant proteolysis of apoE in conditioned medium from glia cultures (Figure 5*D*). When mixed with hippocampal conditioned medium, glial conditioned medium blocked apoE proteolysis, indicating an inhibition of the extracellular hippocampal apoE degrading protease by a glia secreted factor (Figure 5*D*). Cortical conditioned medium did not impair apoE proteolysis mediated by hippocampal conditioned medium (Figure 5*D*).

### α1-antichymotrypsin/neuroserpin-3 inhibits apoE proteolysis

**Figure 6:**
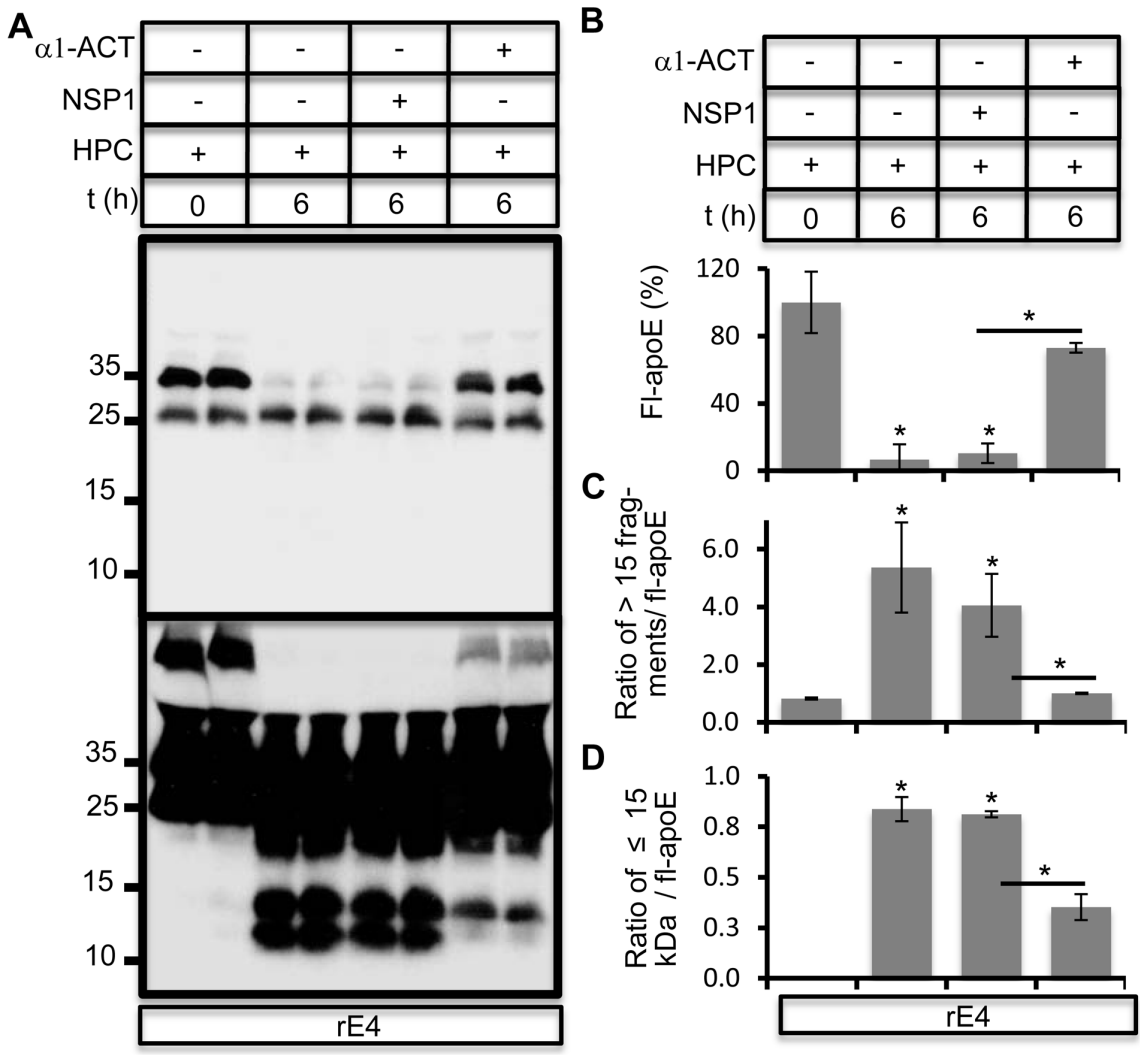
α1-ACT impairs apoE proteolysis. A) rE4 was incubated with hippocampal conditioned medium for 6 hours in absence (ctrl) or presence of either α1-antichymotrypsin (α1-ACT) or neuroserpin-1 (NSP1) at 1 ug/ml. Degradation of apoE was analyzed by western immunoblotting. A longer exposure is in the lower panel. B–D) Levels of full length apoE (B), ratio of apoE fragments greater than 15 kDa (C) and lesser than 15 kDa (D) to full length apoE levels were quantified using ImageJ.

SERPINs (Serine protease inhibitors) are a family of predominantly secreted proteins which possess the ability to inhibit serine proteases. We hypothesized that a member of the SERPIN family such as α1-antichymotrypsin1 α1-ACT) or α1-antitrypsin (AAT) might be responsible for inhibition of chymotrypsin-like protease in the above assay (Figure 5*D*). α1-ACT is a 55–68 kDa member of the SERPIN family expressed in glia cells [38], [39]. Purified human α1-ACT, which is more specific for chymotrypsin-like activity such as chymotrypsin-like protease, cathepsin G and thrombin, significantly inhibited apoE proteolysis in hippocampal conditioned medium. The closely related member of the SERPIN family, neuroserpin-1 (NSP1) that primarily targets serine proteases such as tissue plasminogen activator (tPA), urokinase plasminogen activator (uPA) and plasmin [40], did not inhibit apoE proteolysis (Figure 6*A, 6B*). Appearance of apoE fragments was also significantly impaired in the presence of α1-ACT (Figure 6*C, 6D*).

### Chymotrypsin-like activity in hippocampal conditioned medium and its inhibition by a glia derived factor

**Figure 7:**
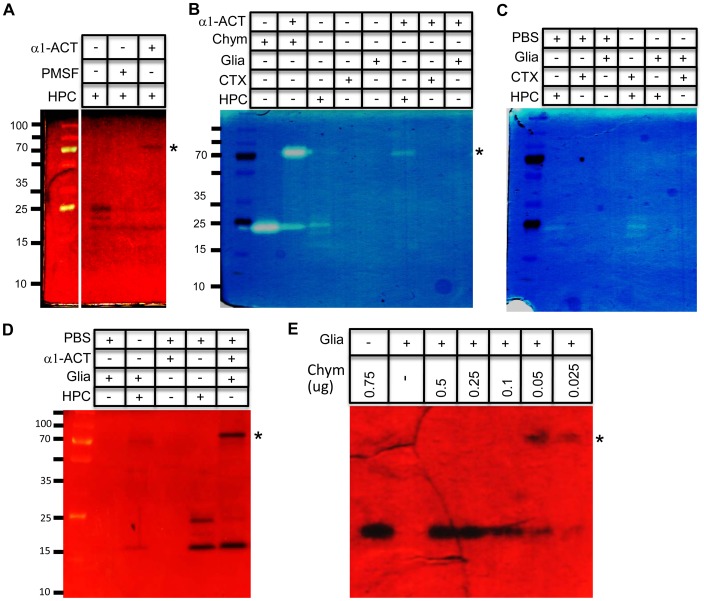
Characterization of chymotrypsin like protease activity and its inhibitor in neuronal and glia conditioned medium respectively. A) DIV 18 hippocampal conditioned medium was incubated in presence or absence of PMSF (1 ug/ml) or 0.25 ug α1-ACT for 2 h at 37°C, followed by casein zymography. B) 0.5 ug α-chymotrypsin (chym) was incubated alone or together with 0.25 ug α1-ACT in PBS for 2 h at 37°C. Similarly, hippocampal (HPC), cortical (CTX) and glia (Glia) conditioned media were incubated alone or together with 0.25 ug α1-ACT for 2 h at 37°C. Samples were later analyzed by casein zymography. C) HPC, CTX and Glia conditioned medium was mixed with PBS or each other in 50∶50 ratio and incubated for 2 h at 37°C and was analyzed by casein zymograpgy. D) Glia conditioned medium was mixed with HPC conditioned medium or PBS as indicated in 90∶10 ratio and incubated 2 h at 37°C followed by casein zymograpgy. Hippocampal conditioned medium alone or pre-incubated with α1-ACT was loaded as control. E) Decreasing amounts of α-chymotrypsin (chym) was incubated with conditioned glia medium as indicated for 2 h at 37°C and was analyzed by casein zymograpgy.

In order to define the proteolytic activities released in neuronal conditioned medium, we performed casein zymography. In hippocampal conditioned medium we identified three major protease activities close to 25 kDa in size (Figure 7*A*). Pre-incubation of conditioned medium for two hours with PMSF or α1-ACT reduced proteolytic activities of two of these activities, supporting their identities as serine proteases. The addition of α1-ACT also caused the appearance of a higher molecular weight proteolytic activity at ∼70 kDa. This activity may represent a complex between active protease and α1-ACT [41], [42]. Purified bovine α-chymotrypsin showed proteolytic activity at 25 kDa, corresponding to its molecular weight (Figure 6*B*). Incubation of α-chymotrypsin with rec α1-ACT for two hours caused a reduction in chymotrypsin activity as expected, and we also observed formation of a similar high molecular weight active complex (Figure 7*B*).

Unlike in hippocampal conditioned medium, no proteolytic activities were detected in cortical and glia conditioned medium (Figure 7*B*). As expected, incubation of these conditioned media with α1-ACT did not result in the appearance of any high molecular weight proteolytic activities. On the other hand, incubation of hippocampal conditioned medium with glia conditioned medium significantly decreased proteolytic activities confirming the presence of inhibitory molecules secreted by glia (Figure 7*C*). We initially did not observe the high molecular weight complex between the hippocampal protease and its glia inhibitor as expected. However, increasing the proportion of glial to hippocampal media resulted in incomplete inhibition of proteolytic activities and the appearance of a weak higher molecular weight activity representing enzyme-inhibitor complex (Figure 7*D*). A similar complex between purified α-chymotrypsin and the glial inhibitor was observed only at low concentrations of α-chymotrypsin (Figure 7*E*). These data further support the model of the secretion of an inhibitor by glia responsible for efficient inhibition of the neuron-derived protease.

### Detection of apoE fragments in human brain

**Figure 8:**
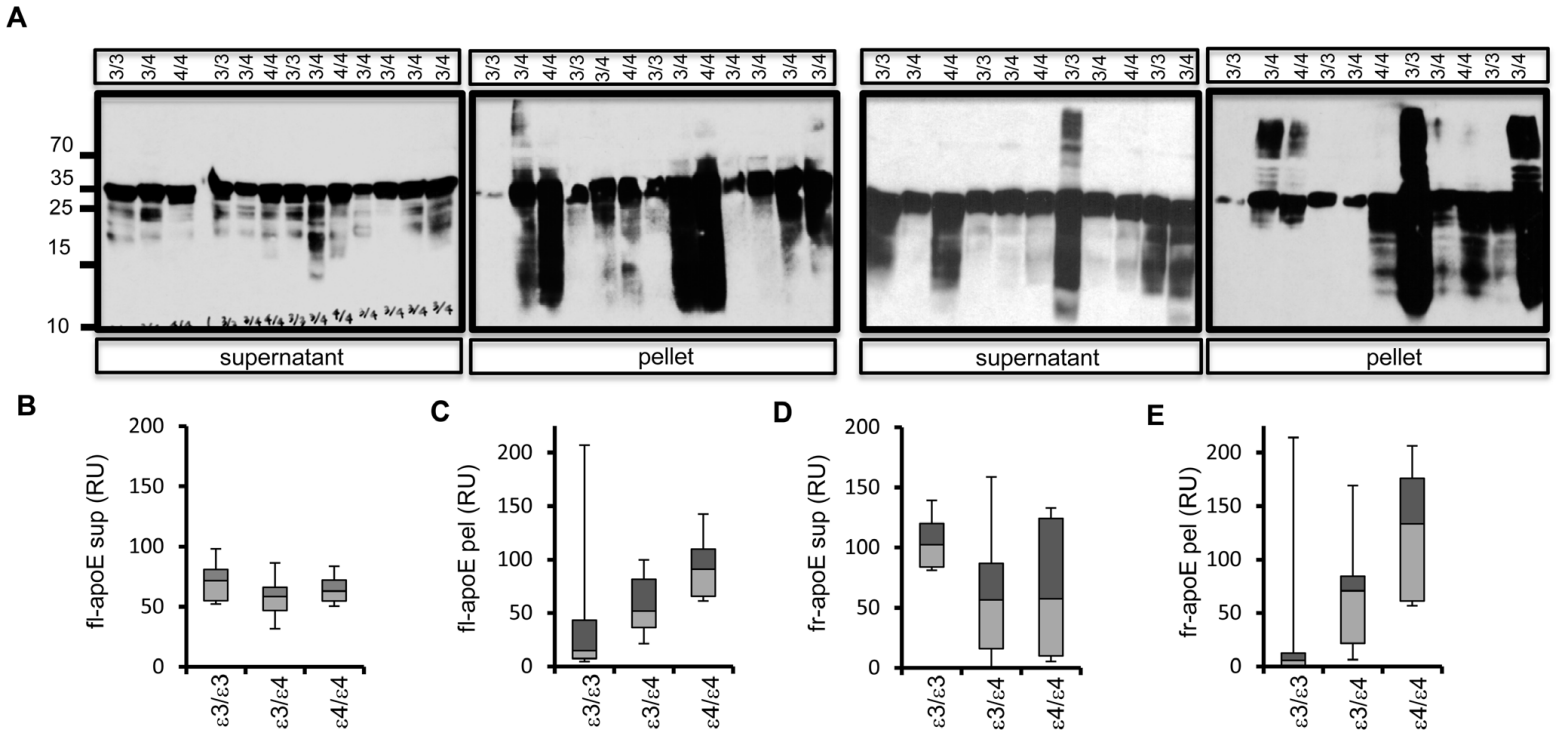
Detection of apoE and its proteolytic fragments in post-mortem human AD brains. A) Frontal cortex tissue obtained from post-mortem human AD brains was genotyped (7 APOE ε3/3; 11 APOE ε3/4, 6 APOE ε4/4) and homogenized to obtain RIPA soluble and insoluble fractions Equal protein from each RIPA (supernatant) and RIPA-SDS (pellet) samples was loaded on SDS-PAGE. ApoE was detected by western immunoblotting soluble and insoluble fractions. APOE genotype for each sample is indicated on top. B–E) Relative levels/units (RU) of full length apoE (fl-apoE) (B, C) and collective lower molecular weight apoE fragments (fr-apoE) (D, E) in supernatant (sup), pellet (pel) were estimated using ImageJ and data was plotted as whiskers diagram (box plots) as indicated.

Previous studies have addressed apoE proteolytic fragments in the presence of AD pathology in human brains [21], [29], [30], [43]. To investigate the effect of APOE genotype on this apoE proteolysis, we analyzed apoE and its fragments in human AD post-mortem brain tissue obtained from subjects with APOE3/E3, APOE3/E4 and APOE4/E4 genotypes. ApoE was detected by western immunoblotting in RIPA (supernatant) and RIPA-SDS (pellet) fractions. Full length apoE (34 kDa) was the predominant species in most brain supernatant and pellet fractions, although lower molecular weight apoE fragments were also abundant in many samples (Figure 8*A*, Figure S6). Whisker diagram analysis of full length apoE in supernatent (Figure 8*B*), pellet (Figure 8*C*) and apoE fragments in supernatent (Figure 8*D*) and pellet (Figure 8*E*) suggests increased accumulation of apoE fragments dependent on APOE-ε4 dose in pellet fractions. These data further highlight that apoE undergoes proteolytic processing in AD brains and apoE fragments are abundant in diseased brains.

## Discussion

Results presented here identify a role of a secreted neuronal serine protease in extracellular metabolism of apoE. Conditioned medium obtained from both hippocampal and cortical cultures mediates apoE proteolysis, generating a pattern of apoE fragments similar to that seen in live neuronal cultures. Less activity was observed from cortical neurons compared to hippocampal neurons. The apoE cleaveage sites and inhibition profile supports a role of chymotrypsin-like protease in extracellular apoE cleavage. We also found a crucial role of glia in regulation of apoE proteolysis.

Several different enzymes, such as cathepsin D, thrombin, chymotrypsin-like serine protease and aspartic proteases, have been proposed to mediate apoE cleavage [29], [30], [35]. We used several approaches to characterize the extraneuronal apoE proteolytic activity in our assay. Its identity as a chymotrypsin serine protease was demonstrated by the complete inhibition of apoE proteolysis by PMSF. Specific inhibitors of thrombin, cathepsin D, and cathepsin G failed to block apoE proteolysis. Inhibition was achieved by the chymotrypsin inhibitor, α1-ACT. A similar apoE proteolytic pattern by purified chymotrypsin also supported our conclusion. Finally, mass spectrometry analysis of apoE fragments identified the presence of peptides generated by chymotrypsin like activity. The initial cleavage of apoE begins with higher specificity chymotrypsin like activity (which cleaves at the carboxy terminal of FYW, but not before P), and probably proceeds with lower specificity chymotrypsin activity (which cleaves at the carboxy terminal of FYWML). Since the mass spectrometry approach depended on experimental generation of trypsin fragments, it is likely that apoE peptides corresponding to other cleavage sites were not seen. Non-chymotrypsin sites in lower molecular weight apoE fragments suggests a contribution of other proteases in subsequent apoE proteolysis.

Earlier studies described identification and partial purification of chymotrypsin-like protease as an intraneuronal protease responsible for apoE cleavage [35]. The molecular weights of some proteolytic fragments observed in those studies resemble the major fragments were observed in our studies. In agreement with earlier studies [21], [32], our data also confirm a neuronal specificity of apoE proteolysis. Previous studies showed generation of apoE fragments in primary cultured neurons and not in astrocytes from transgenic mice expressing apoE in respective cell types [21], [32]. ApoE fragments were detected in brains of mice expressing apoE in neurons (NSE-apoE) and not in astrocytes (GFAP-apoE) [21]. In our experiments, zymography identified three major active proteases close to 25 kDa present in hippocampal neuronal conditioned medium, two of which were inhibited by PMSF and α1-ACT. Serine proteases such as chymotrypsin-like protease (CTRL), chymotrypsin C (CTRC), cathepsin G have molecular weights in this range [44]–[46].

Reduced apoE proteolysis in cortical conditioned medium might result from less expression or inefficient secretion of protease, but reduced apoE proteolysis in glial conditioned media results from the presence of serine protease inhibitor blocking apoE proteolysis. SERPINS such as α1-ACT or AAT1 secreted by glia might be responsible for this effect. In support of this idea we detected active enzyme-inhibitor complexes between the hippocampal conditioned medium protease and the glia conditioned medium inhibitor that corresponded in size to a complex between α1-ACT and purified α-chymotrypsin. Glia are responsible for most apoE biosynthesis and lipidation [47]. A role of glia in regulation of apoE proteolysis further underscores their importance in apoE metabolism and diseases linked with APOE.

The abundance of SERPINs such as α1-ACT and AAT1 might be responsible for the low levels of apoE fragments in healthy human and mice brain, CSF and plasma. Supporting this hypothesis, co-incubation of human plasma or CSF together with hippocampal neuronal conditioned medium did not induce apoE proteolysis (not shown), whereas incubation of VLDL-apoE purified from human plasma was cleaved efficiently (Figure S4). Conformational differences or masking of cleavge site within CSF apoE might also be responsible for its inefficient proteolysis. Alternatively, apoE glycosylation and lipidation can influence susceptibilty of apoE to undergo proteolysis [48], [49]. Both glycosylation and lipidation of apoE is known to impact its interaction with Aβ [50], [51]. On the other hand, pathogenic mechanisms leading to dysregulation of equillibrium between extracellular serine proteases and their inhibitors might contribute to increased levels of apoE fragments in diseased brains. α1-ACT, a SERPIN involved in clearance of proteases via receptor mediated endocytosis [52], is implicated in AD risk and Aβ metabolism, and is found in almost all amyloid plaques [38], [39], [52], [53]. Both α1-ACT and apoE have been shown to respond to pathogenic stressors such as inflammation and can bind to Aβ to promote is polymerization into filaments [54]. ApoE itself may be involved in regulation of α1-ACT [55]. We and others have identified a role of apoE in regulating inflammation [56]–[58]. With the importance of inflammatory cascades in neurodegenerative processes [59]–[61], it will be necessary to elucidate this interplay between apoE proteolysis, α1-ACT and inflammation, and its effects on AD neuropathology.

The presence of apoE fragments in AD brains within amyloid plaques or insoluble fractions has been reported [31], [33], [34], [62], [63]. Findings vary, possibly due to different extraction methods and differential antibody specificity. However, a common finding among these studies is an increase in insoluble full length apoE as well as apoE fragments in AD brains, with more fragments in APOE4 carriers [29], [35], [43], [64]. We also observed a trend towards increased levels of apoE fragments in insoluble fractions in APOE4 carriers. Our in vitro data suggest that apoE4 is metabolized faster to generate lower molecular weight fragments. Structural studies predicted increased susceptibility of apoE4 to proteolysis [65], and pulse-chase experiments showed enhanced degradation and reduced half-life of apoE4 compared to apoE3 in primary astrocytes [24]. Additional mechanisms such as fragment stability, solubility, and interaction of apoE with proteases or inhibitors might contribute to the higher levels of apoE4 fragments.

ApoE directed therapeutics remain an important research area for treating most cases of AD [21], [66]–[70]. ApoE proteolytic fragments have been shown to induce tau phosphorylation and mitochondria dysfunction, leading to behavioral deficits in transgenic mice [32], [35], [71]. Based on these findings, preventing apoE proteolysis has been proposed as a viable drug target against AD [21]. Other targets include the opposing approaches of increasing total apoE levels to compensate for loss of function by apoE4, and decreasing total apoE levels to reduce toxic gain of fuction of apoE4 [23]. Using apoE derived apoE mimetic peptides [72]–[74], modulating the interaction between Aβ and apoE [75], promoting receptor mediated endocytosis of the apoE-Aβ complex [76] or changing apoE4 structure to more closely resemble apoE3 [77] are some of the other proposed therapeutic options against AD associated neurodegeneration. For the success of any of these approaches, it is imperative to understand apoE metabolism and the neuron-glia crosstalk in this process. Identification of the neuron-secreted protease that cleaves apoE in the extracellular space and its regulation by glia is a crucial step in that direction. Development of specific modulators of this process to either increase or decrease apoE proteolysis will provide an opportunity to directly modulate apoE levels.

## Supporting Information

Figure S1 **Metabolism of recombinant apoE by hippocampal neurons.** Rec apoE4 protein (2 ug) was incubated with DIV22 primary hippocampal neurons for indicated time periods and full length apoE and its proteolytic products in conditioned medium as well as cellular lysates were analyzed by western immunoblotting. Cell pellet was lysed in 200 uls RIPA buffer and 20 ul lysate (1/10th) was loaded on gel, whereas 25 uls of total 1 ml (1/40 th) conditioned medium was loaded per sample. Time in hours, t (h) is indicated on top while a longer exposure of the same blot is shown in the right panel. (for 0 h time period apoE was put on the neuronal cultures, followed by quick harvesting of cells and conditioned medium). At t = 0, in addition to full length apoE at 34 kDa, we also detected a ∼25 kDa apoE band and low amounts of higher molecular weight aggregates. More than 50% of full length apoE was metabolized within 6 h of incubation on hippocampal neurons, with almost 80% degradation within 48 h.
(JPG)

Figure S2 **Comassie staining of purified recombinant apoE.** Purified rec apoE4 (rE4) protein (4 ug) and rec apoE3 (rE3) protein (0.7 ug) were loaded on SDS-PAGE and detected by comassie staining.
(JPG)

Figure S3 **Stability of recombinant apoE in non-conditioned control culture medium.** Commercially obtained rE3 and rE4 (2 ug each) were incubated with control hippocampal culture medium for indicated time periods followed by analysis using western immunoblotting. Time in hours, t(h) is indicated on top while a longer exposure of the same blot is shown in the right panel. ApoE levels remain unaltered during incubation and lower molecular weight apoE proteolytic fragments do not appear even after longer exposure.
(JPG)

Figure S4 **Metabolism of human VLDL-apoE by secreted neuronal protease.** Commercially obtained VLDL-apoE (2 ug) purified from human plasma was incubated for indicated times at 37°C with DIV22 conditioned hippocampal medium. ApoE proteolysis apoE was analyzed by western immunoblotting. Time in hours, t(h) is indicated on top.
(JPG)

Figure S5 **Efficient inhibition of thrombin mediated apoE degradation by argotroban.** rE3 and rE4 (2 ug each) were incubated with purified human thrombin (5 U) in presence and absence of 10 uM argotroban (AGTB) as indicated for 24 h followed by analysis using western immunoblotting. Time in hours, t(h) is indicated on top while a longer exposure of the same blot is shown in the right panel. Almost complete degradation of apoE occurred within 24 h by thrombin, with very efficient inhibition of both apoE3 and apoE4 degrdation by 10 uM argotroban.
(JPG)

Figure S6 **Shorter exposures of apoE blots from** **figure 8** **.**
(JPG)

Table S1 Table describes the human AD brains used for the study. BRC# represents the assigned number to each specimen by John Hopkins ADRC Brain Bank.
(PDF)
