# Supplementary figures and images for: Extracellular Proteolysis of Apolipoprotein E (apoE) by Secreted Serine Neuronal Protease
Source: PLoS One. 2014 Mar 27;9(3):e93120. doi: 10.1371/journal.pone.0093120 (PMC3968057; doi:10.1371/journal.pone.0093120)

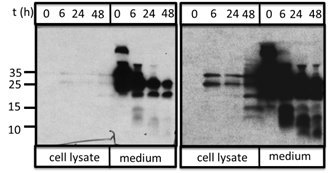

Supplement: Figure S1 — Metabolism of recombinant apoE by hippocampal neurons. Rec apoE4 protein (2 ug) was incubated with DIV22 primary hippocampal neurons for indicated time periods and full length apoE and its proteolytic products in conditioned medium as well as cellular lysates were analyzed by western immunoblotting. Cell pellet was lysed in 200 uls RIPA buffer and 20 ul lysate (1/10th) was loaded on gel, whereas 25 uls of total 1 ml (1/40 th) conditioned medium was loaded per sample. Time in hours, t (h) is indicated on top while a longer exposure of the same blot is shown in the right panel. (for 0 h time period apoE was put on the neuronal cultures, followed by quick harvesting of cells and conditioned medium). At t = 0, in addition to full length apoE at 34 kDa, we also detected a ∼25 kDa apoE band and low amounts of higher molecular weight aggregates. More than 50% of full length apoE was metabolized within 6 h of incubation on hippocampal neurons, with almost 80% degradation within 48 h. (JPG) [file pone.0093120.s001.jpg]

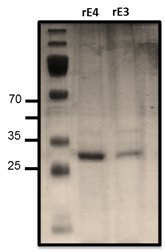

Supplement: Figure S2 — Comassie staining of purified recombinant apoE. Purified rec apoE4 (rE4) protein (4 ug) and rec apoE3 (rE3) protein (0.7 ug) were loaded on SDS-PAGE and detected by comassie staining. (JPG) [file pone.0093120.s002.jpg]

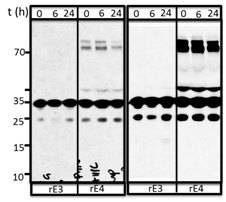

Supplement: Figure S3 — Stability of recombinant apoE in non-conditioned control culture medium. Commercially obtained rE3 and rE4 (2 ug each) were incubated with control hippocampal culture medium for indicated time periods followed by analysis using western immunoblotting. Time in hours, t(h) is indicated on top while a longer exposure of the same blot is shown in the right panel. ApoE levels remain unaltered during incubation and lower molecular weight apoE proteolytic fragments do not appear even after longer exposure. (JPG) [file pone.0093120.s003.jpg]

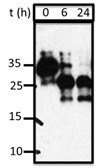

Supplement: Figure S4 — Metabolism of human VLDL-apoE by secreted neuronal protease. Commercially obtained VLDL-apoE (2 ug) purified from human plasma was incubated for indicated times at 37°C with DIV22 conditioned hippocampal medium. ApoE proteolysis apoE was analyzed by western immunoblotting. Time in hours, t(h) is indicated on top. (JPG) [file pone.0093120.s004.jpg]

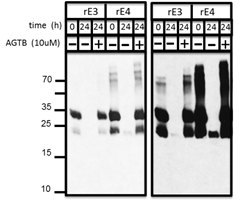

Supplement: Figure S5 — Efficient inhibition of thrombin mediated apoE degradation by argotroban. rE3 and rE4 (2 ug each) were incubated with purified human thrombin (5 U) in presence and absence of 10 uM argotroban (AGTB) as indicated for 24 h followed by analysis using western immunoblotting. Time in hours, t(h) is indicated on top while a longer exposure of the same blot is shown in the right panel. Almost complete degradation of apoE occurred within 24 h by thrombin, with very efficient inhibition of both apoE3 and apoE4 degrdation by 10 uM argotroban. (JPG) [file pone.0093120.s005.jpg]

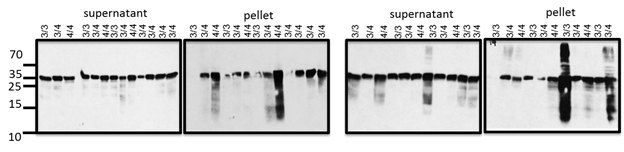

Supplement: Figure S6 — Shorter exposures of apoE blots from figure 8 . (JPG) [file pone.0093120.s006.jpg]
