# Supplementary material for: Extracellular Proteolysis of Apolipoprotein E (apoE) by Secreted Serine Neuronal Protease
Source: PLoS One. 2014 Mar 27;9(3):e93120. doi: 10.1371/journal.pone.0093120 (PMC3968057; doi:10.1371/journal.pone.0093120)
Supplement: Table S1 — Table describes the human AD brains used for the study. BRC# represents the assigned number to each specimen by John Hopkins ADRC Brain Bank. (PDF) [file pone.0093120.s007.pdf]

BRC #	Diagnosis	Age	Sex	Race	PMD	Brain region	Genotyping result
**0276**	AD	82	F	W	8	MFG	ε3/ε3
0364	AD	89	F	W	7	MFG	ε3/ε4
0448	AD	72	F	B	20	MFG	ε4/ε4
0461	AD	61	M	W	20	MFG	ε3/ε3
0697	AD	80	F	W	0	MFG	ε3/ε4
0769	AD	72	M	W	7	MFG	ε2/ε4
0771	AD	73	M	W	6	MFG	ε3/ε4
0901	AD	83	F	W	6	MFG	ε3/ε4
0954	AD	61	F	W	7	MFG	ε3/ε4
0973	AD	78	F	W	8	MFG	ε4/ε4
0992	AD	62	F	W	12	MFG	ε3/ε4
1004	AD	63	F	W	11	MFG	ε3/ε3
1655	AD	92	F	W	8	MFG	ε3/ε4
1663	AD	63	M	W	9	MFG	ε3/ε4
1671	AD	77	M	W	32	MFG	ε4/ε4
1678	AD	92	F	B	13	MFG	ε3/ε4
1695	AD	95	F	W	4	MFG	ε3/ε4
1705	AD	79	F	W	7	MFG	ε3/ε3
1710	AD	90	F	W	5	MFG	ε3/ε4
1821	AD	85	F	W	18	MFG	ε3/ε4
1823	AD	87	F	B	16	MFG	ε3/ε4
1829	AD	94	F	W	4	MFG	ε3/ε4
1866	AD	74	F		5.5	MFG	ε3/ε4
1898	AD	79	M	W	10.5	MFG	ε4/ε4
1935	AD	72	F	W	19	MFG	ε3/ε3
2059	AD	76	M	W	15	MFG	ε3/ε4
2060	AD	77	M	W	20	MFG	ε4/ε4
2136	AD	92	F	W	7	MFG	ε3/ε3
2153	AD	69	M	W	10.5	MFG	ε4/ε4
2273	AD	62	F	W	22	MFG	ε4/ε4
